# A relationship between bruxism and orofacial-dystonia? A trigeminal electrophysiological approach in a case report of pineal cavernoma

**DOI:** 10.1186/1744-9081-9-41

**Published:** 2013-10-28

**Authors:** Gianni Frisardi, Cesare Iani, Gianfranco Sau, Flavio Frisardi, Carlo Leornadis, Aurea Lumbau, Paolo Enrico, Donatella Sirca, Enrico Maria Staderini, Giacomo Chessa

**Affiliations:** 1 Epochè “Orofacial Pain Center”, Rome, Italy; 2 Department of Oral Rehabilitation, University of Sassari, Sassari, Italy; 3 Department of Neurology, Santo Eugenio Hospital, Rome, Italy; 4 Institute of Clinical Neurology, University of Sassari, Sassari, Italy; 5 Department of Anesthesiology, Tor Vergata University, Rome, Italy; 6 Department of Medical-Surgical Speciality, University of Sassari, Sassari, Italy; 7 Neuropharmacology Department, University of Sassari, Sassari, Italy; 8 Western Switzerland University of Applied Sciences, HEIG-VD, Yverdon-les-Bains, Switzerland

**Keywords:** Bruxism, Orofacial pain, Temporomandibular disorders, Dystonia, Oro facial dystonia, Trigeminal electrophysiology, Bilateral Root-MEPs

## Abstract

**Background:**

In some clinical cases, bruxism may be correlated to central nervous system hyperexcitability, suggesting that bruxism may represent a subclinical form of dystonia. To examine this hypothesis, we performed an electrophysiological evaluation of the excitability of the trigeminal nervous system in a patient affected by pineal cavernoma with pain symptoms in the orofacial region and pronounced bruxism.

**Methods:**

Electrophysiological studies included bilateral electrical transcranial stimulation of the trigeminal roots, analysis of the jaw jerk reflex, recovery cycle of masseter inhibitory reflex, and a magnetic resonance imaging study of the brain.

**Results:**

The neuromuscular responses of the left- and right-side bilateral trigeminal motor potentials showed a high degree of symmetry in latency (1.92 ms and 1.96 ms, respectively) and amplitude (11 mV and 11.4 mV, respectively), whereas the jaw jerk reflex amplitude of the right and left masseters was 5.1 mV and 8.9 mV, respectively. The test stimulus for the recovery cycle of masseter inhibitory reflex evoked both silent periods at an interstimulus interval of 150 ms. The duration of the second silent period evoked by the test stimulus was 61 ms and 54 ms on the right and left masseters, respectively, which was greater than that evoked by the conditioning stimulus (39 ms and 35 ms, respectively).

**Conclusions:**

We found evidence of activation and peripheral sensitization of the nociceptive fibers, the primary and secondary nociceptive neurons in the central nervous system, and the endogenous pain control systems (including both the inhibitory and facilitatory processes), in the tested subject. These data suggest that bruxism and central orofacial pain can coexist, but are two independent symptoms, which may explain why numerous experimental and clinical studies fail to reach unequivocal conclusions.

## Background

Dystonia is an involuntary, repetitive, sustained (tonic), or spasmodic (rapid or clonic) muscle contraction. The spectrum of dystonias can involve various regions of the body. Of interest to oral and maxillofacial surgeons are the cranial-cervical dystonias, in particular, orofacial dystonia (OFD). OFD is an involuntary, sustained contraction of the periorbital, facial, oromandibular, pharyngeal, laryngeal, or cervical muscles [1]. OFD can involve the masticatory, lower facial, and tongue muscles, which may result in trismus, bruxism, involuntary jaw opening or closure, and involuntary tongue movement.

The etiology of OFD is varied and includes genetic predisposition, injury to the central nervous system (CNS), peripheral trauma, medications, metabolic or toxic states, and neurodegenerative disease. However, in the majority of patients, no specific cause can be identified. An association was found among painful temporomandibular disorders (TMDs), migraine, tension-type headache, and sleep bruxism, although the association was only significant for chronic migraine. The association between painful TMDs and sleep bruxism significantly increased the risk for chronic migraine, followed by episodic migraine and episodic tension-type headache [2].

Bruxism is the most frequently occurring oral movement disorder, and can occur in subjects while awake and during sleep. Both forms are likely to have different etiologies, and their diagnosis and treatment require different approaches. Treatment is indicated when bruxism causes pain in the masticatory system or leads to damage such as tooth wear or fractures of teeth, restorations, or even of implants. A focused review on the etiology of bruxism [3] concluded that there is a limited role for morphological factors in the etiology of bruxism, while psychological factors (e.g., stress) and pathophysiological factors (e.g., disturbances in central neurotransmitter systems) are more prominently involved.

Orofacial pain (OP), including pain from TMDs, exerts a modulatory effect on mandibular stretch reflexes [4]. Electrophysiological studies have shown that experimentally induced pain from injections of 5% hypertonic saline solution into the masseter muscle causes an increase in the peak-to-peak amplitude of the jaw jerk. This facilitatory effect appears to be related to an increased sensitivity of the fusimotor system, which at the same time causes muscle stiffness [5]. In addition, a number of animal studies of experimentally-induced muscle pain have shown that activation of the muscle nociceptors markedly influences the proprioceptive properties of the muscle spindles through a central neural pathway [6], and that washing of the local algogenic substance causes a return to normal tendon reflexes.

However, few studies have attempted to characterize the pain associated with bruxism (i.e., to examine the neurobiological and physiological characteristics of the mandibular muscles). Some clinical cases and small-scale studies suggest that certain drugs linked to the dopaminergic, serotoninergic, and adrenergic systems can either suppress or exacerbate bruxism. Further, the majority of these pharmacological studies indicate that various classes of drugs can influence the muscular activity related to bruxism, without exerting any effect on OP [7].

Therefore, the sensitization of the trigeminal nociceptive system and the facilitating effect on mandibular stretch reflexes and CNS hyperexcitability are neurophysiopathogenetic phenomena that can be correlated to pain in the craniofacial region. However, up to now, no correlation has been reported between OP, dysfunction of the mesencephalic nuclei, and facilitation of trigeminal nociception, except for a clinical study on a patient affected by pontine cavernoma, which highlighted a relative facilitation of the trigeminal nociceptive system through the blink reflex [8].

Thus, in the present study we performed an electrophysiological evaluation of the excitability of the trigeminal nervous system in a patient affected by pineal cavernoma with pain symptoms in the orofacial region and pronounced bruxism.

## Methods

### Patient description

The subject was a 32-year-old man suffering from pronounced nocturnal and diurnal bruxism and chronic bilateral OP prevalent in the temporoparietal regions, with greater intensity and frequency on the left side. Neurological examination showed a contraction of the masseter muscles with pronounced stiffness of the jaw, diplopia and loss of visual acuity in the left eye, left gaze nystagmus with a rotary component, papillae with blurred borders and positive bilateral Babynski’s, and polykinetic tendon reflexes in all four limbs.

### Magnetic resonance imaging (MRI)

**Figure 1:**
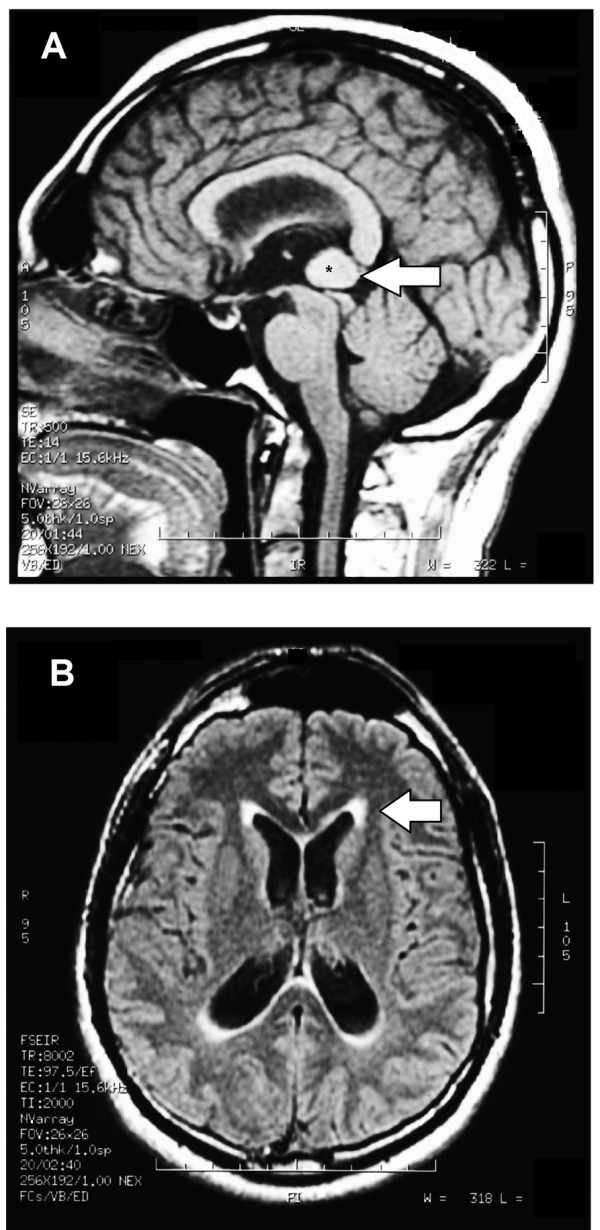
**Magnetic resonance imaging. (A)** Magnetic resonance imaging (MRI) of the brain with contrast medium (gadolinium) the extensive cavernoma can be seen (arrow). **(B)** MRI in the axial plane. The periventricular rim, indicating transependimal liquid absorption, can be seen.

MRI of the brain, using Turbo Spin Echo, Fluid Attenuated Inversion Recovery, and Gradient Echo sequences, was conducted before and after intravenous administration of contrast medium. Results showed the presence of a roundish area of approximately 1.5 cm in diameter located in the vicinity of the quadrigeminal cistern at the level of the pineal gland (Figure 1A). There was also a slight dilation of the supratentorial ventricular system, which appeared in the axis and was most evident in the proximity of the temporal horns, with a periventricular rim with a transependymal fluid absorption phenomenon (Figure 1B). The signal characteristics of the formation suggested a provisional diagnosis of pineal cavernoma.

### Electrophysiological studies

Based on the previous observations, an electrophysiological examination of the patient was performed through bilateral electrical transcranial stimulation of the trigeminal roots, together with a study of the jaw jerk reflex (JJr) and the recovery cycle of the Masseter Inhibitory Reflex (_rc_MIR). In accordance with the Declaration of Helsinki (Helsinki, 1964), written informed consent for the neurophysiological evaluation was obtained from the patient, and the study protocol was approved by the local Ethics Committee. (University of Sassari no. 976).

### Bilateral Trigeminal Root-Motor Evoked Potentials

Bilateral and simultaneous electric transcranial stimulation (_e_TS) of both trigeminal roots elicits a neuromuscular response termed bilateral Root-Motor Evoked Potentials (_b_R-MEPs). This technique was performed using an electromyographic device (NGF-Nemus; EBNeuro, Firenze, Italy) equipped with two electrostimulators. The stimulation electrodes were arranged on the skull as follows: the anode was placed at the vertex and the cathode electrodes were positioned 11–12 cm along the line joining the vertex to the acoustic meatus in the parietal region, on each side [9,10]. The electrical stimulus consisted of a square wave of 250 μs duration, at a voltage of approximately 300 V and a maximum current of 100 mA. The motor potentials evoked after _e_TS of the right and left trigeminal roots were recorded on the right masseter (Ch1) and left masseter (Ch2) muscles through two paired surface electrodes. The electromyographic setting was a 20 ms time-window width, 5 mV per division, and a filter bandwidth of 2–2 kHz.

**Figure 2:**
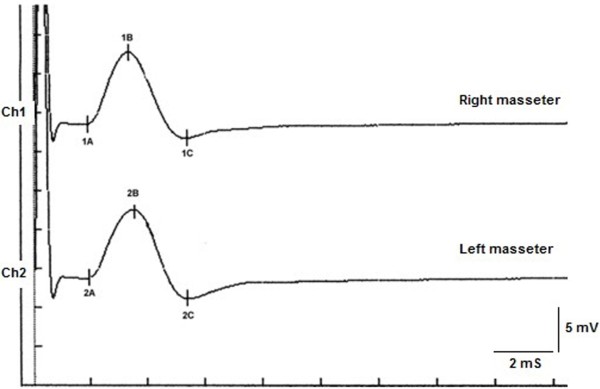
Motor evoked potential of the trigeminal roots.

The peak-to-peak amplitude was also analyzed. The amplitude of the electrical stimulus was maximized to recruit all the trigeminal motor fibers (Figure 2). The peak limit was considered to be reached when an increase in voltage yielded no changes in the amplitude of the muscular response.

### Jaw Jerk reflex

**Figure 3:**
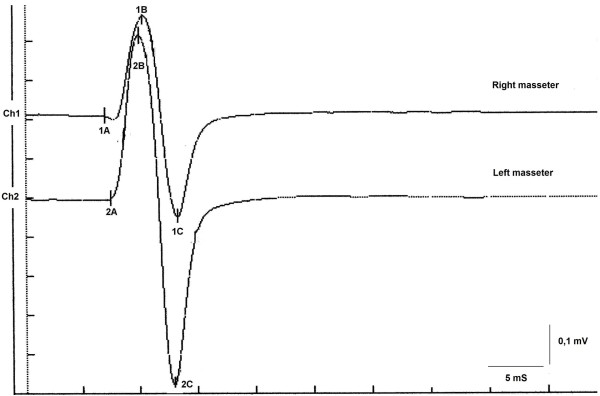
Jaw jerk response recorded on the masseter muscles.

The Jaw Jerk reflex (JJr) was elicited by placing the index finger over the middle of the patient’s chin, and then tapping the index finger with a reflex hammer which triggered recording of EMG activity. The hammer was provided with a piezo-electric force sensor whose signal was acquired on a dedicated channel at the same time with the EMG signals. Although the force of the hit of the hammer was not calibrated, nevertheless we measured the mean force on many trials and only the trials obtained with a force of the hammer within +-10% of the mean force were retained for further processing and averaging. So we may say that all the trials used for averaging were obtained with a hit force of the hammer within 10% of a mean force which, by the way, was not calibrated so we cannot state the exact value in newton. The hit of the hammer was used as trigger for starting trial acquisition. The subject held his jaw in a very slightly clenched intercuspal position. He was then asked to perform five maximal clenches, each lasting up to 3 s, with the mandible held in the intercuspal position to obtain the mean EMG value at maximal voluntary contraction (MVC). During the JJr test, the subject was guided by visual feedback to ensure that the EMG levels were maintained at approximately 20% of the MVC. Electromyographic signals were recorded simultaneously (50 ms time-window width, 100 V per division, filter bandwidth 50–1 kHz) using surface electrodes on the right and left masseter muscles, using an electromyographic device (NGF-Nemus; EBNeuro, Firenze, Italy). The JJr was averaged over 20 trials, and the peak-to-peak amplitude was measured (Figure 3). The evoked jaw jerk responses, the onset latency and peak-to-peak amplitude, and the relationship in amplitude between the JJr and the corresponding amplitude of the ipsilateral R-MEPs are shown as amplitude ratio percentages in Table 1.

**Table 1:** Description of the neuromuscular responses of the jaw jerk response (JJr), the root-motor evoked potentials (R-MEPs) and the amplitude ratio

**Parameters**	**Traces (Channel)**	**R-MEPs**	**JJr**	**Amplitude ratio JJr *vs *** **R-MEPs (%)**
Onset Latency (ms)	1	1.92	7	
	2	1.96	7.5	
Amplitude (ms)	1	11	5.1	46
	2	11.4	8.9	78

The evoked JJr and R-MEPs responses with the onset latency and peak-to-peak amplitude and the relationship in amplitude between the jaw jerk and the corresponding amplitude of the ipsilateral R-MEPs as amplitude ratio percentages. Ch1: right masseter. Ch2: left masseter.

### Recovery cycle of Masseter Inhibitory Reflex

The recovery cycle of the Masseter Inhibitory Reflex (_rc_MIR) was studied by generating pairs of stimuli with identical characteristics, performed percutaneously with a bipolar electrical stimulator positioned on the patient’s face in the area of the mental nerve. The stimulation was produced using square wave electrical impulses, capable of evoking a clearly defined inhibitory reflex composed of two silent periods (SPs), termed SP1 and SP2, separated by an EMG activity recovery interval “Interposed Activity” (IA). The first stimulus (S1) was considered as the conditioning stimulus and the second (S2) as the test stimulus. The inter-stimulus interval between S1 and S2 was set to 150 ms.

**Figure 4:**
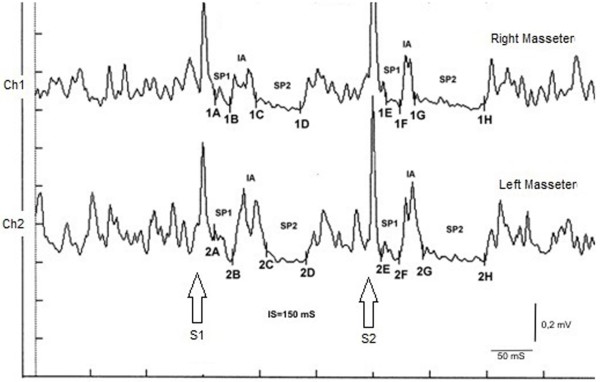
**Recording of the recovery cycle of the Masseter Inhibitory Reflex (**_**rc**_**MIR).** The number of the markers correspond to the EMG traces or channel (1= Ch1= right masseter and 2= Ch2= left masseter); while letters to the sequence of the latencies; SP1 to the first silent period, SP2 to the second silent period and IA to the interposed activity. S1 and S2 correspond to the conditioning stimulus and test stimulus, respectively.

The subject was instructed to clench his teeth to produce the maximum EMG activity and to maintain the contraction for at least 3 s, with the help of visual and audio feedback. After 60 s of rest, the subject repeated the contraction. The EMG signal was recorded in a directly rectified and mediated mode. The positioning of the recording electrodes was the same as that used for recording the JJr, and the pre-amplifier parameters were a 500 ms time-window width, 200 V per division, and a filter bandwidth of 50–1 kHz. The latencies and the durations of the SPs and the IA (Figure 4) were calculated as follows:

i. To simplify the examination, the _rc_MIR was evoked by electrically stimulating the left side only. The EMG responses correspond to the EMG traces of the right masseter (Ch1) and of the left masseter (Ch2). Thus, on the traces, each marker indicates the channel number, while the letters indicate the sequences of the latencies.

ii. The S1 stimulus divides the acquisition into pre- and post-analysis, and generates the SPs and the IA.

iii. The S2 stimulus given to 150 ms after the S1, termed inter-stimulus (IS), evokes the second sequence of SPs and the IA.

iv. The SPs by S1 and S2 are determined automatically by the software that positions the markers on the first and last minimum value processed on the traces for generating the SP1 and SP2, and consequently calculates the duration. The IA duration is calculated between the last minimum value of SP1 and first minimum value of the SP2.

## Results

### _b_R-MEPs

The results are shown in Table 1. Normal results were obtained for both the onset latency, with a difference between the right and left masseter muscles of 40 μs (1.92 ms and 1.96 ms, respectively), and the peak-to-peak amplitudes, with a difference between the right and left masseter muscles of 400 μV (11 mV and 11.4 mV, respectively). This test enabled us to confirm that the trigeminal motor system had not suffered organic damage from compression by the vascular malformation. The perfect symmetry also allowed us to confirm that when the trigeminal system is directly elicited through bilateral electrical transcranial stimulation, it generates a bilateral neuromuscular output that is extremely symmetrical both in conduction speed and in the recruitment of the motor fibers.

### JJr

Conduction along the reflex path was found to be normal (Table 1), with a slight asymmetry of latency between the sides, within normal limits. The recorded amplitude for the right and left masseter was 5.1 mV and 8.9 mV, respectively. In particular, the peak-to-peak amplitude of the JJr was 46% for the right masseter and 78% for the left masseter of the average amplitude of the respective ipsilateral R-MEPs for the masseters. These results were higher than the normal value of 30% [11] (Table 1, amplitude ratio).

### _rc_MIR

In the subject tested the S2 stimulus was able to evoke both SPs, while in a normal subject the S2 was able to evoke a SP1 only, or at most, an SP2 of reduced duration. As shown in Table 2, the duration of the SP1 evoked by S2 was very stable, with no significant differences in the duration of the SP1 generated by S1 (Δ= -1ms for Ch1 and Δ= -2 ms for Ch2) while the SP2 evoked at the right and left masseter by S2 (61 ms and 54 ms, respectively) was longer than that evoked by S1 (39 ms and 35 ms, respectively). The differences were of +22 ms for the Ch1 (right masseter) and +19 ms for the Ch2 (left masseter). Consequently, the duration of the IA showed clear differences between S2 and S1. The duration of the IA evoked by S2 was 12 ms *vs.* 23 ms by S1 for the right masseter (Ch1) and 17 ms *vs* 30 ms by S1 for the left masseter (Ch2) with a difference between the responses evoked by S2 minus S1 of -11 ms and -13 ms, respectively.

**Table 2:** **Description of the duration of the EMG events by the recovery cycle of the Masseter Inhibitory Reflex (**_**rc**_**MIR)**

**Traces/Ch**	**Area**	**Duration (S1) (ms)**	**Duration (S2) (ms)**	**Δ-Duration S2-S1 (ms)**
1	SP1	13	12	-1
1	IA	23	12	-11
1	SP2	39	61	+22
2	SP1	16	14	-2
2	IA	30	17	-13
2	SP2	35	54	+19

Duration and difference in duration of various events of the masseter inhibitory reflex. The EMG events correspond to: first silent period (SP1), interposed EMG activity (IA), and the second silent period (SP2) evoked by the conditioning stimulus (S1) and by the test stimulus (S2). Ch1: right masseter and Ch2: left masseter.

In Table 3, the latencies 1B and 2B from S1 was 24 mS and 26 mS, respectively, and 1F and 2F from S2 was 24 mS and 27 mS, respectively, showing relative symmetry. The onset latency of SP2 (markers 1C, 2C *vs.* 1G, 2G) determines the end of the IA, and was 47 mS and 56 mS, respectively, when evoked by S1, compared to 37 mS and 44 mS, respectively, for S2.

**Table 3:** **Description of the positioning and measurements of the markers for the recovery cycle of the Masseter Inhibitory Reflex (**_**rc**_**MIR)**

**EMG traces**	**Markers**	**Onset Latency (S1) (ms)**	**Markers**	**Onset Latency(S2) (ms)**
Ch1	1A	11	1E	12
	1B	24	1F	24
	1C	47	1G	37
	1D	86	1H	98
Ch2	2A	10	2E	13
	2B	26	2F	27
	2C	56	2G	44
	2D	91	2H	98

Latency value of markers corresponding to the right masseter (Ch1) and left masseter (Ch2). For further explanations please refer to Figure 4 and text.

## Discussion

The main aim of this study was to electrophysiologically document hyperexcitability of the trigeminal nervous system in a patient affected by pineal cavernoma with pronounced symptoms of OP and bruxism, and who was resistant to any pharmacological or odontological treatment.

We found evidence of activation and peripheral sensitization of the nociceptive fibers, the primary and secondary nociceptive neurons in the CNS, and the endogenous pain control systems, including both the inhibitory and facilitatory processes in our subject.

The concentration of extracellular glutamate in 13 patients affected by cavernous angioma [12] was reported to be increased in comparison with physiological concentrations. High levels of glutamate can cause negative effects on the brain through excitotoxic mechanisms, including degeneration of the superficial layer of the retina in a mouse after repeated administration of glutamate, termed “glutamate excitotoxicity” [13], resulting from NMDA receptor hyperactivation [14]. In a study in which the trigeminal ganglion neurons were exposed to KCl, the calculated release of glutamate was 10 times greater than the basal level [15]. Further, a significant reduction in the release of potassium-induced glutamate was observed with addition of ω-agatoxin TK, a powerful P/Q calcium channel blocker, while the N-type calcium channel blocker ω-Cgtx conotoxin had a similar effect [16]. Nimodipine, an L-type calcium channel blocker, was also found to reduce the amount of potassium-induced glutamate release [17]. These studies suggest that the P/Q-, N-, and L-type calcium channels each mediate a significant fraction of depolarization-associated glutamate release.

Glutamate release is obviously a much broader and more complex phenomenon. NMDA, kainate, and AMPA ionotrophic receptors, and the metabotropic glutamate receptors, have been found in the superficial lamina of the trigeminal nucleus caudalis in mice [18]. NMDA and AMPA receptor antagonists can block the transmission of the nociceptive trigeminal-vascular signals [19,20] and reduce the high level of c-*fos* observed in the trigeminal nucleus caudalis following cisternal injection of capsaicin [21]. Furthermore, micro-injections of ω-agatoxin into the ventrolateral area of the periaqueductal gray cause a facilitatory response of nociceptive activity in the trigeminal nucleus caudalis (TNC) activated by tonic electrical stimulation of the supratentorial parietal dura, adjacent to the middle meningeal artery [22]. This response can occur through antinociceptive and/or pronociceptive effects, because the presence of P/Q-type calcium channels is required at the synaptic level for the presynaptic action potentials to couple with the neurotransmitter release processes [23]. Of note, the pre-synaptic afferents in the PAG are positioned on GABAergic inhibitory interneurons and on descending projection neurons. Therefore, the facilitatory effect may be explained by an increased release of GABA, which would indirectly disinhibit the dorsal horn neurons, or by a direct pronociceptive mechanism [24]. These experimental results provide further understanding of the clinical manifestations of pain and central nervous system hyperexcitability found in cases of cerebral cavernous malformations.

Indeed, a blink reflex study on a 38-year-old patient with right hemicranial symptoms associated with a pontine cavernoma affecting the nucleus raphes magnus area revealed a reduction of the pain threshold and a persistent facilitation of the R2 response, with an onset latency difference of 4.4 ms less in the side displaying the symptoms [8]. This confirms a regulatory role for release of neurotransmitters by the nucleus raphes magnus, which exhibits a descending inhibitory control on the TNC [25] and on the entire antinociceptive mesencephalic complex [26]. Our results suggest a hyperexcitability of the trigeminal nervous system in our subject, as follows. First, we evoked a direct response of the trigeminal motor system (_b_R-MEPs) to provide a value for reference and for amplitude symmetry, as the direct response of the trigeminal motor branch was not affected by any conditioning. A comparison between the jaw jerk responses versus the ipsilateral responses of the R-MEPs showed a much higher amplitude ratio than in normal subjects [11] (Table 1). Therefore, these data indicate the presence of hyperexcitability of the trigeminal system.

The facilitatory effect on the masseter reflex could be indirect. The highest concentration of premotoneurons in the orofacial motor nuclei is found in the bulbar and pontine reticular formations adjacent to the motor nuclei themselves, where these are GABAergic, glycinergic, and glutamatergic-type premotoneurons [27]. In addition, the significant increase of the SP2 recovery cycle from S2 compared with the response from S1 (Table 2) corroborates the hypothesis of hyperexcitability of the trigeminal system. In an *in vitro* study performed on encephalic slices [28], intracellular recording of interneurons of the peritrigeminal area (PeriV) surrounding the trigeminal motor nucleus (NVmt) and of the parvocellular reticular formation (PCRt) demonstrated that electrical stimulation of the adjacent areas could evoke both excitatory postsynaptic potentials (EPSPs) and inhibitory postsynaptic potentials (IPSPs). All the EPSPs induced by stimulation of the PeriV, PCRt, and NVmt were shown to be sensitive to ionotropic glutamate receptor antagonists (DNQX and APV), while the IPSPs were sensitive to the GABA and glycine receptor antagonists, bicuculline and strychnine. The cells of this sample showed a long after-hyperpolarization (AHP).

In an electrophysiological study that analyzed a population of neurons and interneurons in the NVmt [29], three types of AHP were seen: fast, slow, and biphasic. The majority of the motoneurons had a fast AHP (fAHP), whereas most of the interneurons had a slow AHP. The basic properties of these interneurons are similar to the previously described “last-order pre-motoneurons” in the PeriV [30], suggesting that the interneurons in the NVmt are part of an interneuronal matrix surrounding the NVmt in which the motoneurons are inserted. In this last study, the authors describe the possibility, although rare, of interneurons also having an fAHP.

In our study, the increased duration of the SP2 from S2 invades the IA rather than expanding into the EMG reactivation after the silent period. The afferents for the SP2 descend their intra-axial process along the trigeminal spinal tract and connect with a polysynaptic chain of excitatory interneurons located in the reticular formation at the level of the pontocerebellar junction. The last interneuron in the chain is inhibitory, and sends ipsilateral and controlateral collaterals that ascend medially to the right and left spinal trigeminal complex to reach the trigeminal motoneurons [31]. The interneural sensitization in the _rc_MIR may be linked to a combination of the excitatory effect of glutamate, with a contribution from the intraneuronal fAHP, and to the disinhibition of the inhibitory processes due to the effect of glycine and GABA.

Overall, our data suggest that certain types of OP, at least those of a central origin, and bruxism are caused by a disruption and homeostatic imbalance of cerebral neurobiochemistry, particularly of the excitatory and inhibitory neurotransmitters in the trigeminal nervous system.

This gives rise to the following questions: Is there a correlation between OP and bruxism, and can bruxism be considered a clinical form of orofacial dystonia?

With respect to the correlation, a distinction should be made between central and peripheral OP on the basis of case history and clinical examination. The muscle discomfort of bruxism is mainly a peripheral phenomenon, resulting from muscle hyperfunction leading to destruction of the myofibrils and release of algogenic substances including myoglobin into bloodstream. By contrast, OP radiating to one or more areas of the face correlated with a clear manifestation of nocturnal or diurnal bruxism could be considered a central type disorder. In these cases, trigeminal electrophysical examinations are highly informative, particularly the _rc_MIR, blink reflex, JJr, and _b_R-MEPs, for a differential diagnosis between organic-type lesions of the CNS and functional-type diseases such as TMDs.

Thus, although bruxism and central OP can coexist, they are two independent symptoms, which is why many experimental and clinical studies fail to reach unequivocal conclusions [32].

It is also possible that bruxism may be a clinical form of dystonia. Our data indicate that bruxism may be a clinical manifestation linked to a CNS neurotransmitter imbalance, and therefore should be considered a subclinical condition of orofacial dystonia or dystonic syndrome. Nevertheless, this phenomenon also appears in a transitory form in children and is resolved with the eruption of mixed dentition [33,34].

Many studies and diagnostic research protocols, including the Research Diagnostic Criteria (RDC), continue to appear in the field of OP and TMDs, although clear consensus has not yet been reached among the international scientific community [35]. The RDC should consider the patient as affected by a painful syndrome, and should tend towards the definition of a differential diagnosis between organic and/or functional pathologies [36].

## Abbreviations

bR-MEPs: bilateral root-notor evoked potentials; JJr: Jaw jerk reflex; rcMIR: recovery cycle of masseter inhibitory reflex; SP2: Silent period 2; S2: Test stimulus; S1: Conditioning stimulus; OFD: Orofacial dystonia; OP: orofacial pain; TMDs: Tempomandibular disorders; IA: Interposed activity; NVmt: Trigeminal motor nucleus; PeriV: Peritrigeminal area; PCRt: Parvocellular reticular formation; EPSPs: Excitatory postsynaptic potentials; IPSPs: Inhibitory postsynaptic potentials; AHP: After-hyperpolarization.

## Competing interests

The authors declare that they have no competing interests.

## Authors’ contributions

GF and FF drafted the first manuscript and contributed to data acquisition and interpretation. GF, FF, GFS and EMS supervised the study, participated in its design and coordination, and revised the manuscript that led to the final approval of the current submission. CI is a treating neurologist of the patient, and made a contribution to data acquisition and interpretation. GC, CL, PE, DS, and AL contributed to acquisition and interpretation of data literature search. All authors read and approved the final manuscript.
